# 464. Whole Genome Sequencing and Mutation Analysis of SARS-CoV-2 in the Immunocompromised Patients with Persistent SARS-CoV-2 Shedding

**DOI:** 10.1093/ofid/ofad500.534

**Published:** 2023-11-27

**Authors:** Eui Jin Chang, Jungmin Lee, Sung-Woon Kang, So Yun Lim, Ji Yeun Kim, Choi-Young Jang, Man-Seong Park, Sung-Han Kim

**Affiliations:** Department of Internal Medicine, Asan Medical Center, Seoul, Korea, Seoul, Seoul-t'ukpyolsi, Republic of Korea; Korea University College of Medicine, Seoul, Seoul-t'ukpyolsi, Republic of Korea; Asan Medical Center, Seoul, Seoul-t'ukpyolsi, Republic of Korea; Asan Medical Center, Seoul, Seoul-t'ukpyolsi, Republic of Korea; Asan Medical Center, Seoul, Seoul-t'ukpyolsi, Republic of Korea; Asan Medical Center, Seoul, Seoul-t'ukpyolsi, Republic of Korea; Korea University, Seoul, Seoul-t'ukpyolsi, Republic of Korea; Asan medical center, Seoul, Seoul-t'ukpyolsi, Republic of Korea

## Abstract

**Background:**

Immunocompromised individuals could be sources of new SARS-CoV-2 variants as the virus can reside in them for extended periods, acquiring new mutations. This study aims to investigate the mutations acquired by SARS-CoV-2 in the immunocompromised with persistent viral shedding during the Omicron era.

**Methods:**

From February to November 2022, we enrolled immunocompromised adults within 12 weeks from the initial SARS-CoV-2 diagnosis. Nasopharyngeal swabs, saliva, and blood samples were obtained weekly. SARS-CoV-2 polymerase chain reaction, viral culture, plaque reduction neutralization test, and whole-genome sequencing were conducted for at least two samples in each patient.

**Results:**

Thirteen patients were included in the final analysis. Eleven patients (84.6%) had hematologic malignancy and two (15.4%) got solid organ transplantation. Nine patients (69.2%) were identified by WGS analysis as initially having BA.2 or BA.2.3 sub-lineages. They acquired five nonsynonymous mutations as the median during the median period of 51 days. The S region had the highest proportion of acquired mutations associated with immune evasiveness and other variants of concern. One patient acquired a mutation associated with resistance against remdesivir, ORF1b:V792I, after receiving several cycles of remdesivir. Despite high concentrations of neutralizing antibodies, another patient showed persistent viral shedding, possibly due to the emergence of an immune-evasive mutation, S:L452Q.

Changes of nonsynonymous mutations in sequenced SARS-CoV-2 genomes in each patient over time and COVID-19 treatments

**Figure:**
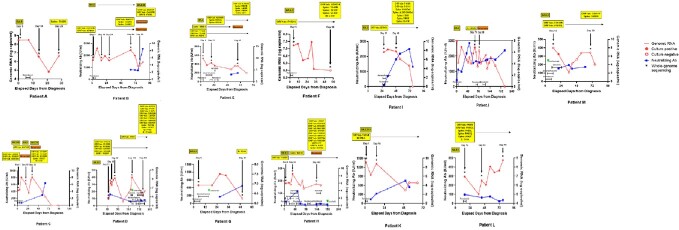

Cumulative numbers of all the nonsynonymous mutations in sequenced SARS-CoV-2 genomes in each patient over time

**Figure:**
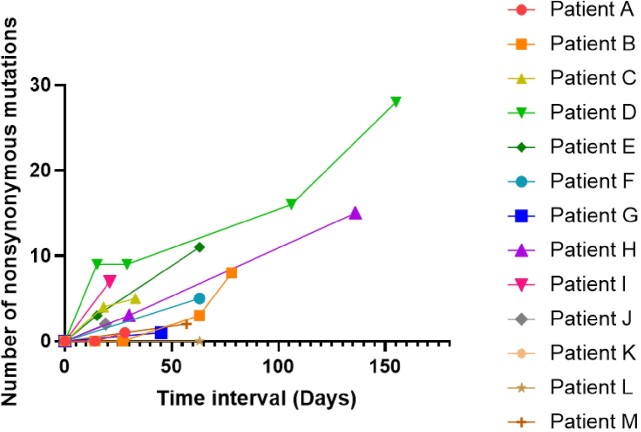

The first-day WGS analysis performed for each patient was considered day 0.

Characteristics of acquired nonsynonymous mutations in the SARS-CoV-2 genome from each immunocompromised patient compared with the initial nearest SARS-CoV-2 lineage

**Figure:**
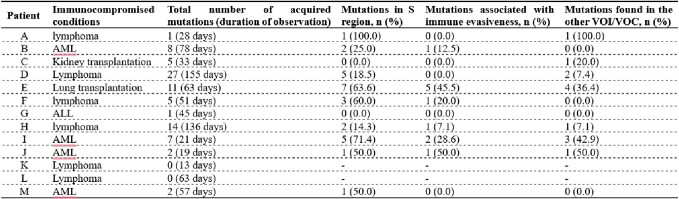

**Conclusion:**

The immunocompromised with SARS-CoV-2 could be the source for the emergence of new variants with immune evasiveness or remdesivir resistance. Ending isolation for the immunocompromised with SARS-CoV-2 should be cautiously determined due to the potential for transmission of viruses with new mutations.

Acquired nonsynonymous mutations which were found as the defining mutations of the other VOIs or VOCs

**Figure:**
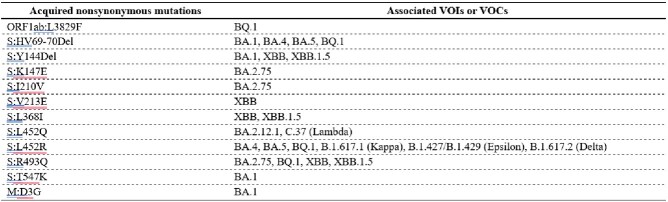

Clinical characteristics of the immunocompromised patients

**Figure:**
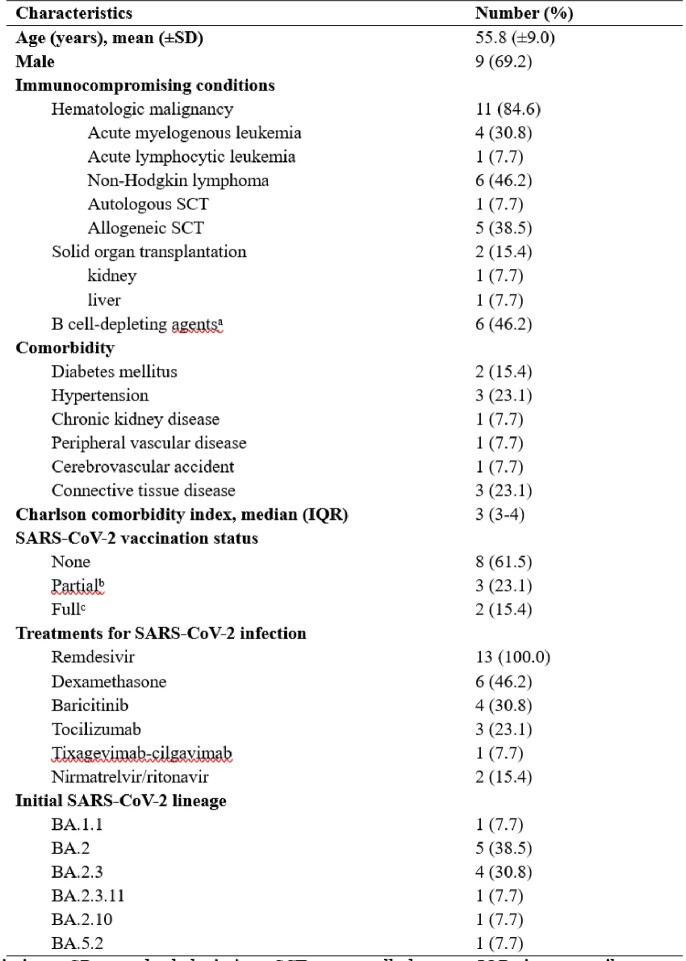

Abbreviations: SD, standard deviation; SCT, stem cell therapy; IQR, interquartile range; SARS-CoV-2, severe acute respiratory syndrome-coronavirus-2 a Use of anti-CD20 monoclonal antibodies or bispecific T cell engagers within two years b One or two vaccinations against SARS-CoV-2 with Comirnaty®, Spikevax®, or Vaxzevria® c More than two vaccinations against SARS-CoV-2 with Comirnaty®, Spikevax®, or Vaxzevria®

Comparison of the clinical differences between the immunocompromised who acquired mutations relatively more rapidly and slowly

**Figure:**
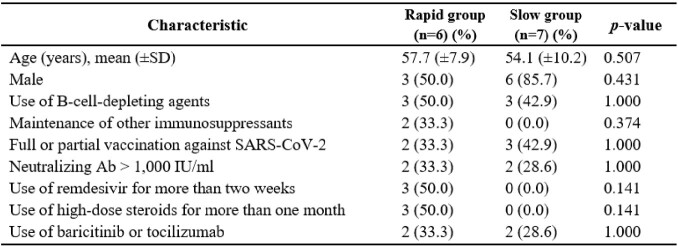

**Disclosures:**

**All Authors**: No reported disclosures

